# Effects of Defatted Rice Bran–Fortified Bread on the Gut Microbiota Composition of Healthy Adults With Low Dietary Fiber Intake: Protocol for a Crossover Randomized Controlled Trial

**DOI:** 10.2196/59227

**Published:** 2024-08-29

**Authors:** Hwei Min Ng, Jasjot Maggo, Catherine Louisa Wall, Simone Brigit Bayer, Warren C McNabb, Jane Adair Mullaney, Meika Foster, Diana L Cabrera, Karl Fraser, Janine Cooney, Tania Trower, Catrin S Günther, Chris Frampton, Richard Blair Gearry, Nicole Clemence Roy

**Affiliations:** 1 Department of Medicine University of Otago, Christchurch Christchurch New Zealand; 2 High-Value Nutrition National Science Challenge Auckland New Zealand; 3 Riddet Institute Massey University Palmerston North New Zealand; 4 AgResearch Grasslands Palmerston North New Zealand; 5 Edible Research Christchurch New Zealand; 6 Plant and Food Research Palmerston North New Zealand; 7 Plant and Food Research Ruakura Research Centre Hamilton New Zealand; 8 Biostatistics and Computational Biology Unit University of Otago, Christchurch Christchurch New Zealand; 9 Department of Human Nutrition University of Otago Dunedin New Zealand

**Keywords:** dietary fiber, defatted rice bran, bread, healthy adults, gut microbiota, metabolites, gut physiome, randomized controlled trial, mobile phone

## Abstract

**Background:**

Inadequate dietary fiber (DF) intake is associated with several human diseases. Bread is commonly consumed, and its DF content can be increased by incorporating defatted rice bran (DRB).

**Objective:**

This first human study on DRB-fortified bread primarily aims to assess the effect of DRB-fortified bread on the relative abundance of a composite of key microbial genera and species in fecal samples. Secondary outcomes include clinical (cardiovascular risk profile), patient-reported (daily bread consumption and bowel movement, gut comfort, general well-being, and total DF intake), biological (fecal microbiota gene abundances, and fecal and plasma metabolites), and physiome (whole-gut and regional transit time and gas fermentation profiles) outcomes in healthy adults with low DF intake.

**Methods:**

This is a 2-armed, placebo-controlled, double-blinded, crossover randomized controlled trial. The study duration is 14 weeks: 2 weeks of lead-in, 4 weeks of intervention per phase, 2 weeks of washout, and 2 weeks of follow-up. Overall, 60 healthy adults with low DF intake (<18 g [female individuals] or <22 g [male individuals] per day) were recruited in Christchurch, New Zealand, between June and December 2022. Randomly assigned participants consumed 3 (female individuals) or 4 (male individuals) slices of DRB-fortified bread per day and then placebo bread, and vice versa. The DRB-fortified bread provided 8 g (female individuals) or 10.6 g (male individuals) of total DF, whereas the placebo (a matched commercial white toast bread) provided 2.7 g (female individuals) or 3.6 g (male individuals) of total DF. Before and after each intervention phase, participants provided fecal and blood samples to assess biological responses; completed a 3-day food diary to assess usual intakes and web-based questionnaires to assess gut comfort, general and mental well-being, daily bread intake, and bowel movement via an app; underwent anthropometry and blood pressure measurements; and drank blue food dye to assess whole-gut transit time. Additionally, 25% (15/60) of the participants ingested Atmo gas-sensing capsules to assess colonic gas fermentation profile and whole-gut and regional transit time. Mean differences from baseline will be compared between the DRB and placebo groups, as well as within groups (after the intervention vs baseline). For metabolome analyses, comparisons will be made within and between groups using postintervention values.

**Results:**

Preliminary analysis included 56 participants (n=33, 59% female; n=23, 41% male). Due to the large dataset, data analysis was planned to be fully completed by the last quarter of 2024, with full results expected to be published in peer-reviewed journals by the end of 2024.

**Conclusions:**

This first human study offers insights into the prospect of consuming DRB-fortified bread to effectively modulate health-promoting gut microbes, their metabolism, and DF intake in healthy adults with low DF intake.

**Trial Registration:**

Australian New Zealand Clinical Trials Registry ACTRN12622000884707; https://www.anzctr.org.au/Trial/Registration/TrialReview.aspx?id=383814

**International Registered Report Identifier (IRRID):**

DERR1-10.2196/59227

## Introduction

### Background

Diet strongly influences the gut microbiota composition [1-3]. Diets low in dietary fibers (DFs) may contribute to gut dysbiosis, where specific bacterial taxa and microbial diversity are diminished [1,2]. This phenomenon mostly occurs in industrialized populations consuming a low-DF, high–fat and protein diet [3]. However, alteration of the gut microbiota profile may be reversible by improving DF intake [2].

DFs are carbohydrates that are neither digestible nor absorbable in the small intestine. Some DFs are fermented by gut microbes in the large intestine, which produces metabolites such as organic acids [4]. These metabolites, in particular the short-chain fatty acids acetate, butyrate, and propionate, play major beneficial physiological and immunological roles for the human host [4]. They modulate the luminal pH and immune system, lower pathogenic bacteria load, act as a catalyst for calcium and magnesium absorption, and provide energy sources for colonic cells [4]. Therefore, increasing DF intake may provide the required substrates for microbial growth and energy, ultimately benefiting the human host’s health [2].

DFs are found in a wide range of plant foods, yet inadequate DF intake is still a ubiquitous issue in the adult population worldwide [5-12]. Fortification of commonly consumed foods with synthesized or extracted DF may improve the DF intake of adult populations [2]. Bread is the main source of DF worldwide [6,10,12,13] and is one of the oldest foods that offer health benefits to humans [13]. Bread is commonly consumed by all cultures and ethnicities and is a staple food for many individuals. Furthermore, bread is ideal for incorporating ingredients such as cereal brans to increase DF content [14,15], which may modulate the gut microbiota.

Rice bran is a cheap cereal by-product of rice milling [14]. Rice bran has a purported hypo-allergenicity and has a unique nutrient profile rich in DF, protein, antioxidants, and minerals as well as phytochemicals [13,16-18]. Only a few human studies have been undertaken on rice bran [19-36]. These studies have focused on the use of modified arabinoxylan (a phytochemical) rice bran on subjective global assessments in participants with predominant-diarrhea or mixed-type irritable bowel syndrome [19], heat-stabilized rice bran on modulation of the gut microbiota composition [20,22] and metabolites (short-chain fatty acids and bile acids) [20,22] or rice bran fermented using *Lentinus edodes* on natural killer cell activity and cytokine production [30] in healthy adults, rice bran intervention in colorectal cancer survivors [21] or individuals with a high risk of colorectal cancer [23] or cardiovascular diseases [24,26-28,31-36], or hydrolyzed rice bran in patients with cervical cancer [29].

Among these published rice bran studies, only 4 adult human studies have reported the impact of rice bran on the gut microbiota. When compared with baseline values, a daily consumption of 30 g of heat-stabilized rice bran over 2 weeks increased the relative abundance of the genera *Methanobrevibacter*, *Paraprevotella*, *Ruminococcus*, *Dialister*, *Barnesiella*, and *Anaerostipes* and, at 4 weeks, increased the genera *Bifidobacterium* and *Clostridium* [20], whereas 40 g over 24 weeks increased the taxa from the family *Veillonellaceae* in healthy adults [22]. Similarly, in a population at high risk of colorectal cancer, a 24-week intervention of 30 g of rice bran increased the relative abundance of the *Firmicutes* phylum, *Lactobacillus* genus*, Bifidobacterium* genus, and *Prevotella_9* species [23] compared with baseline levels. However, other measures of the microbial community based on the *Firmicutes*-to-*Bacteroidetes* ratio and α-diversity showed mixed effects. Heat-stabilized rice bran reduced the *Firmicutes*-to-*Bacteroidetes* ratio at 2 weeks, with no difference at 4 weeks compared with baseline [21], yet the ratio increased following a 24-week rice bran intervention [23]. While other studies did not report α-diversity [19,20,22], one study found an increased diversity at 4 weeks but not at 2 weeks [21] or at 24 weeks compared with baseline values [23].

The interventions of these studies were either modified arabinoxylan rice bran or heat-stabilized or normal rice bran. They were administered to participants of varied health status and in various forms (powder, rice bran–enriched meals and snacks, and oil) [19-32,34-37]. However, to the best of our knowledge, no human studies have used defatted rice bran (DRB) in bread as an intervention. The process of defatting increases the insoluble fiber content, thereby increasing its proportion of DF [14]. Therefore, several studies have suggested using DRB as a value-added food ingredient [17,38-41]. Taken together, bread, which is commonly eaten by adults, fortified with DRB may help promote beneficial gut microbes and DF intake and, ultimately, provide beneficial health effects to the adult population.

### Hypotheses and Objectives

This study was based on the hypothesis that the consumption of 3 (for female individuals) or 4 (for male individuals) slices of DRB-fortified bread per day for 4 weeks will increase the relative abundance of a composite of key genera and species in fecal samples (proxy of lower gut microbiota) known to be involved in plant glycan metabolism or known to be modulated by the DRB-fortified bread.

Secondary hypotheses were that consumption of DRB-fortified bread would lead to improvements in clinical, patient-reported outcomes; biological measurements; whole-gut and regional transit time; and gas fermentation profiles.

DFs are compounds that require a network of microbes to degrade their complex structures [42,43]. Furthermore, due to the inconsistent findings on the relative abundance of microbial taxa reported by existing rice bran studies, a microbiome composite index is a useful primary outcome for this study.

The primary aim of this study is to determine the influence of the consumption of 3 (for female individuals) or 4 (for male individuals) slices of DRB-fortified bread on the composition of selected genera and species of microbiota that are known to be involved in plant glycan metabolism or known to be modulated by DRB-fortified bread using shotgun metagenomics sequencing of fecal samples.

The secondary aims were to investigate the influence of the consumption of 3 (for female individuals) or 4 (for male individuals) slices of DRB-fortified bread in comparison with placebo white bread on (1) upper- and lower-gut comfort using validated gut-specific questionnaires; (2) mental health and general well-being parameters using validated questionnaires; (3) DF intake using 3-day food diaries; (4) relative abundance of individual taxa, predictive function (gene abundances), and diversity indexes of the fecal gut microbiota using shotgun metagenomic sequencing and of fecal and plasma metabolites using liquid chromatography mass spectrometry (LCMS); (5) cardiovascular risk profile based on anthropometry, blood pressure, and blood lipid concentrations; (6) whole-gut transit time using blue food dye in all participants; and (7) regional and whole-gut transit time and gas fermentation profiles using Atmo gas capsules (Atmo Biosciences) in a subset of participants.

## Methods

### Study Design

This protocol was put together in accordance with the SPIRIT (Standard Protocol Items: Recommendations for Interventional Trials) guidelines for clinical trial protocols [44].

### Study Overview

**Figure 1:**
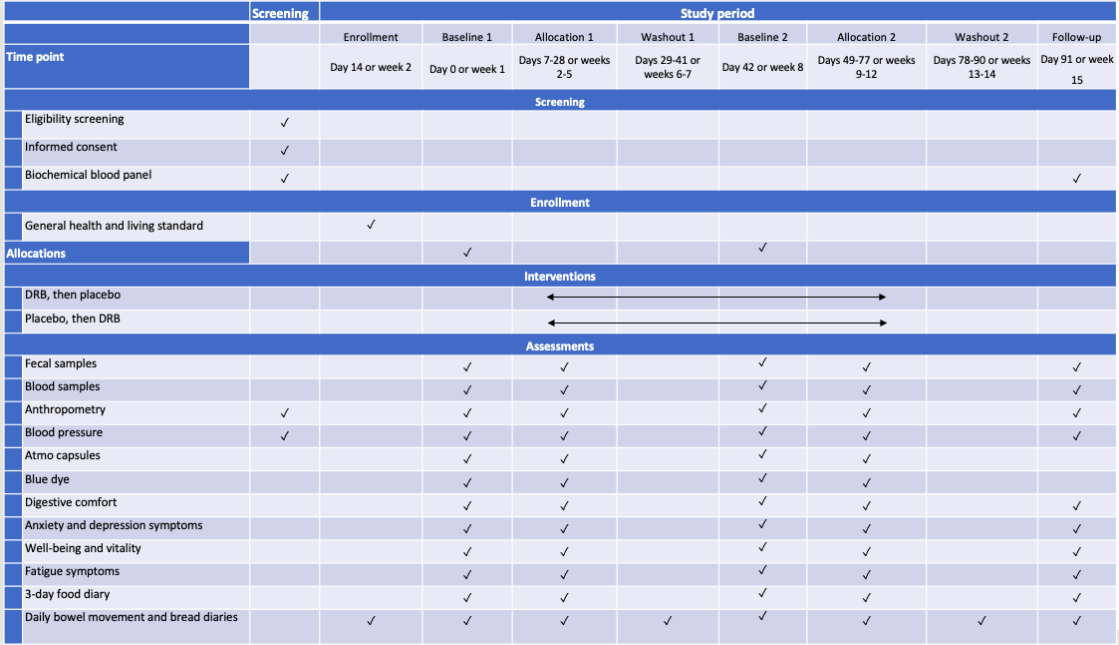
Overview of the schedule of screening, enrollment, interventions, and assessments. DRB: defatted rice bran.

**Figure 2:**
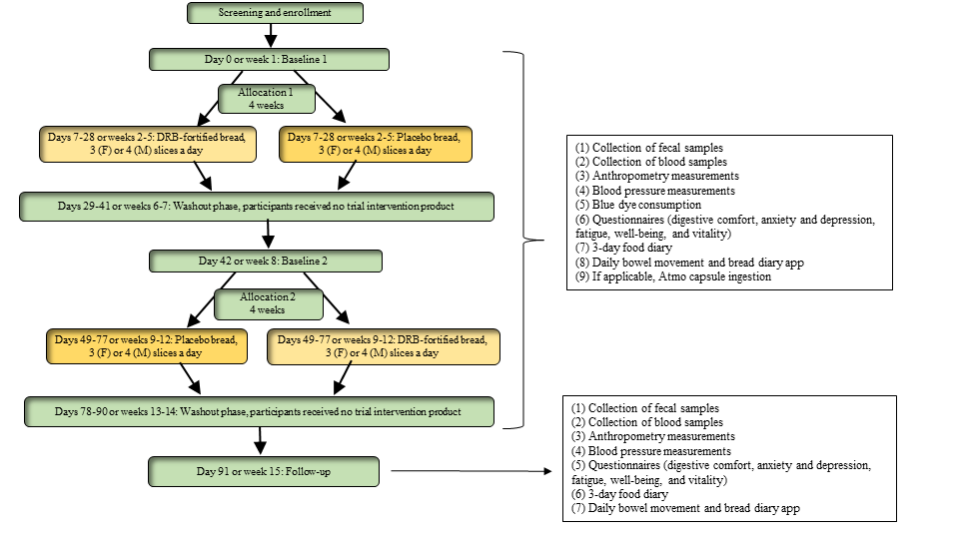
Study flowchart. DRB: defatted rice bran; F: female; M: male.

The Bread Related Effect on Microbial Distribution study was a double-blinded, placebo-controlled, crossover randomized controlled trial of healthy adults with low DF intake. The design and management of the clinical study adhered to the CONSORT (Consolidated Standards of Reporting Trials) guidelines [45]. This design accounts for the recognized variability in individuals and enables each participant to be their own control for their assigned interventions [46,47]. The study duration was a nominal total of 14 weeks, consisting of a 2-week lead-in phase, two 4-week intervention phases separated by a 2-week washout phase, and a final 2-week follow-up phase (Figure 1). During the first intervention period, one group of participants consumed 3 slices (for female individuals) or 4 slices (for male individuals) of DRB-fortified bread daily for 4 weeks, whereas the other group consumed placebo bread. Following a 2-week washout period, participants who consumed the fortified bread in the first period crossed over to consume placebo bread in the second period, and vice versa (Figure 2). Participants were instructed not to alter their diet during the 2-week washout period. The washout period was selected based on gut microbiota studies reporting a return of microbiota abundances to baseline level within 2 weeks after the intervention [48].

Before study commencement, a mutually agreed schedule was discussed and set with each participant to ensure that they understood all expected clinic or visit times and the study timeline. However, when a participant could not come to the clinic on the day a phase was ending, the participant’s needs were accommodated by allowing for earlier visits to the clinic for up to 3 days before the end of the intervention phase (minimum of 25 days of the 28-day intervention). This approach reduced the risk of participants exhausting intervention supplies before the visit and noncompliance or dropout due to scheduling issues.

### Ethical Considerations

The study was carried out in accordance with the International Council for Harmonisation of Technical Requirements for Pharmaceuticals for Human Use, national and local requirements, and the Declaration of Helsinki. All participants gave written, informed consent before taking part in the study. Ethics approval was sought from the University of Otago Human Ethics Committee for Health (H22/061), and consultation took place with the University of Otago, Christchurch, Māori Research Advisor. Before commencement, the study was registered at the Australian New Zealand Clinical Trials Registry (registration ACTRN12622000884707). Recruitment and interventions were conducted at the University of Otago, Christchurch, New Zealand.

The researchers collecting data were experienced in data collection, maintaining security and health-related confidentiality. No identifying or identifiable information about participants was reported in this study, including names, dates of birth, images, or aspects of their circumstances that could identify them. Identifying information of participants was gathered at enrollment, entered by the researchers collecting data, and accessed via REDCap (Research Electronic Data Capture; Vanderbilt University) only by these researchers.

To encourage compliance, enrolled participants were provided with supermarket vouchers worth NZD $300 (US $185; NZD $60, US $37 per visit) upon completing the study. The selected participants for the substudy were also given an additional NZD $100 (US $62) to compensate for their time and inconvenience.

### Protocol Amendments

Any modifications to the protocol that may impact the conduct of the study, the potential benefit to the patients, or patient safety, including changes in study objectives, study design, patient population, sample sizes, study procedures, or substantial administrative aspects, required a formal amendment to the protocol. Any amendments to the study protocol were required to be reported to the University of Otago Human Ethics Committee for Health and all other local approval committees. Changes pertaining to Māori (eg, recruitment processes and analyses) were required to be reported to the Māori Research Advisor. However, the study had no amendments or changes to the protocol.

### Recruitment

Volunteers from the public were recruited through study posters, advertisements translated into Te Reo Māori, social media (web-based presence and Facebook), and word-of-mouth advertisements through Māori health nurses and general practitioners. Interested individuals contacted the research team via email or phone call. They were then sent a patient information sheet, including a consent form, via email, and individuals were provided with sufficient time to consider participating in the study. Once the individual agreed to participate, a web-based screening questionnaire was sent to ascertain their eligibility, DF intake, approximate self-reported BMI, and health conditions.

### Eligibility Criteria

All interested volunteers were assessed for eligibility based on the inclusion and exclusion criteria.

#### Inclusion Criteria

Participants were deemed eligible according to the following criteria: (1) good general health; (2) age between 18 and 65 years; (3) BMI between 18 and 35 kg/m^2^; (4) low DF intake, defined as <22 g per day for male individuals and <18 g per day for female individuals, estimated using a validated habitual DF intake short food frequency questionnaire (see the following sections for further details); (5) no history of bowel disease; (6) nonsmokers; (7) no fiber supplement consumption during the month before screening; and (8) willingness to consume 3 slices (for female individuals) and 4 slices (for male individuals) of bread provided during the intervention periods.

#### Exclusion Criteria

The exclusion criteria were as follows: (1) indication of inability to comply with the study procedures; (2) antibiotic use within the last month; (3) allergies or intolerance to wheat, rice, or gluten; (4) pregnancy, breastfeeding, or plans for a pregnancy in the 3 months after screening or during the study period; (5) alarm features associated with bowel habits, such as recent changes in bowel habits (onset of <3 months), rectal bleeding, sudden weight loss, occult blood in fecal matter, anemia, anal fissures, bleeding hemorrhoids, and family history of gut cancer at an early age; (6) known significant gut disorders and diseases, such as chronic constipation, diarrhea, irritable bowel syndrome, inflammatory bowel disease, diverticulitis, celiac disease, or previous bowel resection; (7) chronic diseases, including cardiovascular disease, cancer, renal failure, previous upper- or lower-gut surgery other than cholecystectomy or appendectomy, and neurological conditions (such as multiple sclerosis, spinal cord injury, or stroke); (8) known systemic conditions, such as heart disease, kidney disease, diabetes, metabolic syndrome, and psychological disorder that could influence the gut directly or through medication use, such as prokinetic, opioid, and opiate or nonsteroidal anti-inflammatory drug use; (9) fasting blood glucose of ≥6.0 mmol/L; and (10) laxative or pre- and probiotic supplement use and indicated inability or unwillingness to stop using 7 days before sample collections.

### Assessment of Low DF

Low DF intake was assessed during the preliminary screening process using a DF intake short food frequency questionnaire [49]. This questionnaire assesses the frequency of fruit, vegetable, bread and cereal, nut and seed, and legume consumption over the previous year. The author of this questionnaire (Dr Genelle Healey) provided a DF calculation spreadsheet to the specified research team members. The raw food group frequency data were entered into the spreadsheet to calculate total DF intake (grams per day).

Participants were grouped as either low, moderate, or high DF intake based on New Zealand gender–specific DF intake cutoffs. The cutoff points were low (<18 g per day for female individuals and <22 g per day for male individuals), moderate (18-24.9 g per day for female individuals and 22-29.9 g per day for male individuals), and high (>25 g per day for female individuals and >30 g per day for male individuals). Only participants meeting the low DF intake cutoffs were included in the study.

### Nonexclusion Criteria

Participants were included in the study if they were diagnosed and had stable health conditions (systemic conditions and diabetes) for >3 months. Selective serotonin reuptake inhibitors, tricyclics, or nonsteroidal anti-inflammatory drugs were permitted if the medication had been continually used and the condition was stable for >3 months.

### Screening

Individuals who fulfilled the inclusion criteria were invited to an on-site full screening visit at research clinics in Christchurch, New Zealand. The screening visit involved a full explanation of the study, health history and dietary habit interviews, collection of written informed consent, standard blood tests (9-hour fast before visit), and blood pressure measurement. Height and weight were used to calculate BMI as body weight (kilograms)/height (meters squared).

Participants who specified being unable to swallow capsules were not considered for the Atmo substudy but were still eligible for the main study.

### Enrollment

#### Questionnaires

Upon being informed of their enrollment into the study, participants were emailed an individualized link to complete 2 web-based enrollment questionnaires. These questionnaires, which were administered via REDCap, took approximately 10 minutes to complete. The Modified Hunter New England Health Survey includes the validated 12-item Short-Form Health Survey version 2 (SF-12v2) and selected question domains from the New South Wales Population Health Survey for diabetes, smoking, alcohol consumption, and physical activity. The SF-12v2 assesses 8 health domains (physical functioning, role—physical, bodily pain, general health, vitality, social functioning, role—emotional, and mental health). This questionnaire was used to assess the general well-being of each participant and raise any important issues. Only the SF-12v2 was scored.

Participants also completed the Economic Living Standard Index Short Form developed and validated by the New Zealand Ministry of Social Development assessing the standard of living and socioeconomic background.

#### Daily Bowel Movement and Daily Bread Diary App

Participants were instructed to download an app titled “BREAD STUDY.” The app consists of both the daily bowel movement and bread diary questions. The questions in the app were captured electronically via the REDCap system using the unique identifier allocated to the participants at the point of consent to the study. The app was designed to be more user-friendly as compared with receiving daily SMS text messages or email links to questionnaires. These daily diaries aimed to obtain a comprehensive record of bowel habits, intervention compliance, and other bread consumption during washout periods. More information pertaining to these questionnaires will be provided later in the *Measurements and Outcomes* section.

### Removal and Withdrawal Criteria

Participation in this study was voluntary, and participants could withdraw at any time without explanation, as stated on the participant information sheet.

In case of the following occurrences, participants were withdrawn:

- The participant requested withdrawal from the study. Participants could decide to end their involvement in the study for any reason and at any time during the study.
- The investigator decided to withdraw a participant from the study when considered necessary. Examples include (1) nonrespect of at least one of the selection criteria after inclusion, (2) noncompliance with the study protocol, and (3) gastroenteritis or antibiotic use.
- The participant reported allergic reactions or adverse effects from either intervention.

Regardless of eligibility or continuing participation, an electronic case report form (eCRF) record was generated for each participant. The eCRF was designed specifically for the needs of this study and was the data collection instrument for the study. Therefore, all data requested on the eCRF were recorded, and all missing data were explained. Each eCRF had a unique 5-digit identifier to link the participant who had completed the screening questionnaire to their own data in a deidentified manner. Collected deidentified data, such as laboratory information, demographic information, and obtained questionnaire data, were stored in a password-protected customized database.

### Termination Criteria for the Whole Study

In case of serious safety concerns, the principal investigators could terminate or interrupt the study. If new information on the risk-to-benefit ratio of the intervention (including treatment or investigational processes) used in the study was obtained in the meantime, the principal investigators reserved the right to interrupt or terminate the project. In addition, premature termination of the study was possible if the principal investigators noticed that participant recruitment was insufficient and could not be accelerated through appropriate measures.

### Study Intervention

The main intervention was bread fortified with DRB. It is a commonly eaten grain and is safe for human consumption [14,50-54]. On the basis of measured composition, the main intervention had 18% of the flour (cereal weight) replaced with 18% of the flour (cereal) weight used in the placebo bread and provided 8 g (for female individuals) and 10.6 g (for male individuals) of total DF. Of the 8 to 10.6 g of DF in 3 to 4 slices of fortified bread, 2.2 g (for female individuals) and 2.9 g (for male individuals) were fiber from wheat, and 5.8 g (for female individuals) and 7.7 g (for male individuals) were from the DRB. The placebo was a matched commercial white toast bread without DRB, providing 2.7 g of DF for female individuals (3 slices) and 3.6 g of DF for male individuals (4 slices). Toast slices were chosen for this study to ensure that the production of DRB-fortified bread had a texture similar to that of the placebo white toast bread while providing the required DF content.

### Blinding and Allocation Concealment

Participants were not informed of the bread ingredients until the completion of the study data collection to maintain blinding. Analysts and researchers were blinded to the order of treatment that the participants received and to which group the participants were assigned during data collection and remained blinded until the statistical analysis of the primary outcome data was completed. The manufacturer of the bread was responsible for labeling both breads to maintain blinding of research team members. To further increase blinding, the manufacturer added colored malt to the placebo bread to make both breads visibly indistinguishable. Specified research team members were responsible for the randomization, enrollment, and data collection of participants; the handout of the intervention and placebo bread; and the management of the stock. Unblinding was only permissible once statistical analysis of the primary outcome data was completed.

### Randomization

One week before the first baseline visit, participants were randomly assigned in a 1:1 allocation using randomized permuted blocks (block size 4) to either DRB-fortified or placebo bread. Randomization was performed by drawing a folded note from a sealed opaque box.

### Measurements and Outcomes

#### Timing of Assessments

Upon enrollment, there were 5 clinic visits in total. Study assessments were conducted at baseline 1 (day 0 or week 1), postintervention assessment 1 (day 29 or week 5), baseline 2 (day 42 or week 8), postintervention assessment 2 (day 78 or week 12), and follow-up (day 91 or week 15). A total of 15 participants who were part of the Atmo substudy ingested capsules at 4 time points: baseline 1 (day 0 or week 1), postintervention assessment 1 (day 29 or week 5), baseline 2 (day 42 or week 8), and postintervention assessment 2 (day 78 or week 12).

#### Biological Measurements

##### Fecal Samples

###### Overview

The primary outcome was the differences in the relative abundance of a composite of selected key genera and species of the gut microbiota known to be involved in plant glycan metabolism or known to be modulated by the DRB-fortified bread (see the *Sample Size* section for more details) as compared with the placebo bread.

Once enrolled, participants received a posted cooler bag containing a fecal collection kit and ice pack. Participants collected a fecal sample into the provided fecal container 24 hours before their clinic visits at baseline, the postintervention time point of each phase, and follow-up. Participants were instructed to label the fecal container with the time and date collected and record it again on the bowel movement diary app. Participants had to refrigerate the collected fecal sample and transport it within 24 hours of sample collection in the provided cooler bag for each clinic visit. If participants could not provide a fecal sample 24 hours before, they were encouraged to collect and bring the sample in the cooler bag as soon as possible following their clinic visits.

In total, 5 aliquots (1 g each) of a fecal sample were frozen for microbial composition, gene abundance, and metabolite analyses. The samples were kept chilled until aliquoted in the laboratory. All aliquots were snap frozen in liquid nitrogen before storage at –80 °C until analysis.

###### Microbial Composition and Gene Abundances

Taxonomic composition and gene abundance of the fecal microbiome were assessed through shotgun metagenomics. Libraries for metagenomics sequencing of the extracted DNA were performed by Auckland Genomics at the University of Auckland, New Zealand. Microbial metagenomic libraries were then generated using the seqWell purePlex DNA library preparation approach. Briefly, genomic DNA was diluted to 5 ng/μL and then sheared and bar-coded with unique dual indexes using transposases to produce a library with fragment sizes of 300 to 1500 base pairs (bp). Libraries were pooled in batches of 24 samples and subjected to a paramagnetic bead-based cleanup. The DNA samples underwent quality control checks for purity and concentration using a bioanalyzer. The samples were shotgun sequenced using the Illumina NovaSeq 6000 platform using 2 × 150 bp paired-end sequencing at Novogene in Singapore.

The quality of the raw data was checked using FastQC (version 0.11.9; Babraham Bioinformatics) [55]. The Trimmomatic software (version 0.36; USADEL LAB) [56] was used for the removal of adapters and low-quality (Phred scores of <30) and short (<36 bp) sequencing reads. Read pairs were aligned to the human reference genome (RefSeq: GCF_000001405) using the “mem” algorithm of BWA (version 0.7.17-r1188) [57], and FASTQ files were generated from the unmapped sequencing reads using the “fastq” function of SAMtools (version 1.8) [58]. Read pairs were joined using PEAR (version 0.9.6) [59] with default settings. Read pairs that did not join were pasted together with a string of Ns using the “fuse” function from the BBMap package (version 38.22-0; Department of Energy Joint Genome Institute) [60]. Joined and fused reads from different lanes from the same sample were compiled into a final “clean” read sample file. Metagenomics functions were obtained through the “blastx” function of DIAMOND (version 0.9.22) [61], mapping the reads against the “nonredundant” National Center for Biotechnology Information database [62]. MEGAN (version 6; ultimate edition) [63] was used to assign putative functions to the alignment files produced by Diamond. This alignment was performed by Paul Maclean (statistician; AgResearch).

Differential relative abundance was performed using *DESeq2* in R (version 1.40.2; R Foundation for Statistical Computing). This package analyzes differential expressed taxa based on a negative binomial distribution. The effect of group was assessed by comparing between groups. The top 10 differentially abundant taxa were extracted based on adjusted *P* values for both baseline and postintervention assessments for each group. Taxa that changed within each group were identified by finding the set difference between the top taxa between baseline and the postintervention assessment. Log2FoldChange values were extracted for the identified changed taxa in each group [64]. Enrichment of functional gene attributes arranged hierarchically as level 1 (broadest level of function, eg, metabolism and cellular processes), level 2 (specific functions, eg, carbohydrate metabolism and amino acid metabolism), and level 3 (detailed pathways, eg, glycolysis and glycan degradation) were analyzed using the R package *Microbiome Multivariable Associations with Linear Models*, which uses general linear models that accommodate crossover designs [65]. Fixed effects included in the model were groups (DRB-fortified vs placebo bread).

###### Metabolome

The relative intensity of fecal metabolites was assessed in the polar, semipolar, and nonpolar fractions of fecal samples using untargeted metabolite profiling (metabolomics).

Fecal samples were freeze-dried under vacuum and extracted using a previously described method [66] with minor modifications. Briefly, 50 mg of lyophilized fecal samples were homogenized with a ceramic bead for 1 minute, and then 400 µL of 75% methanol/Milli-Q water were added. The tubes were vortexed for 30 seconds, sonicated for 2 minutes, and transferred into ice for 10 minutes. Afterward, 1 mL of methyl tert-butyl ether was added, and the mixture was incubated for 1 hour at 450 revolutions per minute. Then, 550 µL of Milli-Q water were added, and after 10 minutes of incubation, the mixture was centrifugated at 14,000 × g for 25 minutes to separate the aqueous (lower) and organic (upper) phases. The polar and lipid extracts were evaporated under a stream of nitrogen at room temperature. The dried samples were stored at –80 °C until LCMS analysis. The metabolite profiling analyses were carried out using high-resolution LCMS on a Shimadzu 9030 quadrupole time-of-flight mass spectrometer equipped with electrospray [67]. An aliquot of the polar extract was taken and analyzed using hydrophilic interaction liquid chromatography [67], and semipolar metabolites were resolved using reverse-phase chromatography [68]. The organic phase was analyzed using the lipidomic methodology [69].

###### Targeted Metabolites

Aliquots of 1 g of fecal samples were sent to Plant & Food Research, New Zealand, for organic acid (including short-chain fatty acids) measurement using an LCMS method. In total, 14 linear and branched organic acids (carbon-1 to carbon-6) were derivatized with a probe using mass spectrometry (MS) probe and stable isotope techniques as previously described [70] with modifications (J Cooney, unpublished data, September 2017) and measured using targeted LCMS on a SCIEX LCMS/MS QTRAP 5500 instrument equipped with a Turbo V ion source and atmospheric pressure chemical ionization probe coupled to an ExionLC ultra–high-pressure liquid chromatography (HPLC) system (Shimadzu). Labeled internal standards for each organic acid were used to ensure accurate quantification [71]. Textbox 1 provides the names and corresponding acronyms of the organic acids analyzed.

**Plasma and fecal organic acids analyzed using liquid chromatography mass spectrometry with the corresponding acronym.**

**Full name and acronym**

- Formic acid: FA
- Lactic acid: LA
- Acetic acid: AA
- Propanoic acid: PA
- Isobutyric acid (2-methylpropanoic acid): IBA
- Butyric acid (butanoic acid): BA
- Succinic acid: SucA
- 2-methylbutyric acid (2-methylbutanoic acid): 2MBA
- Isovaleric acid (3-methylbutanoic acid): IVA
- Valeric acid (pentanoic acid): VA
- 3-methylvaleric acid: 3MVA
- 4-methylvaleric acid (isocaproic acid): 4MVA
- Caproic acid: CA
- Hexanoic acid: HA

Extraction methods for bile acid analysis followed those outlined elsewhere [72]. Briefly, 50 mg of wet fecal samples were extracted with 100 µL ice-cold methanol containing internal standards (10,000 nM of deuterium labelled cholic acid and deuterium labelled chenodeoxycholic acid). The mixture was homogenized for 30 seconds, incubated at –4 °C for 30 minutes, and centrifuged at 18,000 × g for 20 minutes. Then, 20 µL of the supernatant were dissolved in 80 µL of 0.1% aqueous formic acid solution, vortexed, and proceeded to HPLC for analysis. This analysis was carried out using high-resolution LCMS on a SCIEX LCMS/MS QTRAP 6500+ system coupled to an ExionLC system [73]. Textbox 2 provides the names and corresponding acronyms of the bile acids analyzed.

**Fecal bile acids analyzed using liquid chromatography mass spectrometry with the corresponding acronym.**

**Full name and acronym**

- β-muricholic acid: βMCA
- Cholic acid: CA
- Chenodeoxycholic acid: CDCA
- Deoxycholic acid: DCA
- Glycocholic acid: GCA
- Glycohyodeoxycholic acid: GHDCA
- Glycoursodeoxycholic acid: GUDCA
- Glycolithocholic acid: GLCA
- Hyocholic acid: HCA
- Hyodeoxycholic acid: HDCA
- Isolithocholic acid: ILA
- Lithocholic acid: LCA
- Taurine: Taurine
- Tauro-β-muricholic acid: TβMCA
- Tauro-α-muricholic acid: TαMCA
- Taurocholic acid: TCA
- Taurochenodeoxycholic acid: TCDCA
- Taurodeoxycholic acid: TDCA
- Tauroursodeoxycholic acid: TUDCA
- Ursodeoxycholic acid: UDCA

##### Blood Samples

###### Overview

In total, 2 research team members trained in phlebotomy collected peripheral blood at each clinic visit. At both screening and follow-up clinic visits, 1 aliquot (6 mL) of a blood sample was taken into a lithium-heparin vacutainer tube to measure fasting blood glucose, lipid panel (total cholesterol, low-density lipoprotein [LDL] cholesterol, high-density lipoprotein [HDL] cholesterol, and triglycerides), C-reactive protein, and liver function, enzymes, or general health markers, and 1 aliquot (4 mL) of blood was taken into an ethylenediaminetetraacetic acid tube (Becton, Dickinson and Company) for a complete blood count. Before and following each intervention phase, 1 aliquot (6 mL) of a blood sample was taken into a lithium-heparin vacutainer tube (Becton, Dickinson and Company) to analyze the complete lipid profile. The collected blood samples were kept at room temperature and delivered to Canterbury Health Laboratories (Christchurch, New Zealand) for analysis within 1 hour of collection.

At all time points except for the screening clinic visits, an additional 6 mL of blood were collected into 1 × 6-mL lithium-heparin vacutainer tubes to assess plasma metabolome and known metabolites (bile acids and organic acids). The 6-mL vacutainer was kept on ice and processed within 1 hour. The tubes were centrifuged at 4 °C for 5 minutes at 2000 × g, with high acceleration and slowest deceleration to separate plasma from cells. Plasma samples were distributed into 500-µL aliquots and stored at –80 °C until further analysis.

###### Metabolome

Polar metabolites were extracted using a single-phase extraction [68]. Briefly, 50 µL of plasma were extracted using 450 µL of prechilled acetonitrile:water (9:1 v/v). The mixture was shaken for 60 seconds and then centrifuged at 14,000 × g and 4 °C for 10 minutes, and then the extract was placed into an HPLC vial for analysis. Plasma semipolar metabolite extraction was conducted as described previously [74]. Plasma semipolar metabolite extraction was performed by adding 400 µL of ice chloroform to methanol in a 1 to 1 part volume to volume ratio to 50-µL plasma samples, vortexing it for 30 seconds, and incubating it for 1 hour at –20 °C. Then, 200 µL of Milli-Q water were added to the mixture, vortexed, and centrifuged at 14,000 × g and 4 °C for 10 minutes. The supernatants were evaporated to dryness under a stream of nitrogen and stored at –80 °C until analysis. On the day of the analysis, the semipolar extracts were thawed at room temperature (18 °C, –2 °C to +2 °C) and redissolved in redissolved in acetonitrile to water in a 1 to 9 part volume to volume ratio. The mixture was vortexed for 1 minute and then centrifuged (14,000 × g at 4 °C for 10 minutes). The extract was then transferred into an HPLC vial for analysis. Lipid extraction was performed by placing 10 µL of plasma into an Eppendorf tube, adding 95 µL of butanol to methanol in a 1 to 1 part volume to volume ratio, and then spiking it with 5 µL of internal standard SPLASH mix (Avanti Polar Lipids, Merck KGaA) [69]. The mixture was then vortexed for 1 minute, sonicated at room temperature for 60 minutes, and centrifuged at 14,000 × g for 10 minutes at 20 °C. The extract was then transferred to an HPLC vial and stored at –80 °C until LCMS analysis. The plasma polar, semipolar, and nonpolar extracts were analyzed using the LCMS methods for the plasma samples [69].

###### Targeted Metabolites

Textbox 1 shows the names and corresponding acronyms of the organic acids analyzed. Aliquots of 200 μL of heparin plasma were sent to Plant & Food Research, New Zealand, for analysis of organic acids via LCMS. A total of 14 linear and branched organic acids (carbon-1 to carbon-6) were derivatized with a probe using MS probe and stable isotope techniques as previously described [70] with modifications (J Cooney, unpublished data, September 2017) and measured using targeted LCMS on a SCIEX LCMS/MS QTRAP 7500 instrument equipped with a Turbo V ion source and atmospheric pressure chemical ionization probe coupled to a Nexera ultra-HPLC system (Shimadzu). Labeled internal standards for each organic acid were used to ensure accurate quantitation [71].

#### Clinical Measurements

##### Anthropometry

Height, weight, BMI, and waist circumference were measured at each visit (ie, screening, baseline, postintervention assessment, and follow-up) according to the procedures established by the New Zealand Ministry of Health protocols for collecting height, weight, and waist measurements [75].

Standing height was measured to the nearest 0.1 cm using a calibrated stadiometer. Body weight was measured to the nearest 0.1 kg using calibrated body weight scales. Both measurements were taken with participants removing heavy outer clothing and shoes. Waist circumference was taken over clothing, and hence, the level of the measurement was determined by the participant to identify their waist. This measurement was taken to the nearest 0.1 cm using a calibrated measuring tape. All measurements were conducted twice, with a third reading when the 2 readings varied by >0.5 units. BMI was calculated as weight (in kilograms) divided by height (in meters squared).

##### Blood Pressure

Blood pressure was also measured at each morning clinic visit (ie, during screening, at each phase of baseline, at the postintervention assessment, and at follow-up) according to the recommendations established by the Australian expert consensus [76]. The measurement was taken using a validated, automated blood pressure monitor with a fitted-sized upper arm cuff. Participants were seated at rest for 5 minutes with feet flat on the floor, legs uncrossed, back and arm supported in a relaxed position, and the cuff at heart level. Measurements were taken on their nondominant arm. The blood pressure readings were then recorded immediately and compared with those recorded in previous clinic visits. When a high blood pressure value reading was noted, participants were asked about any unusual incidents that may affect the readings. These were then recorded on the eCRF of the participant.

##### Lipid Profile

As mentioned previously, the full lipid profile was measured at each morning clinic visit (ie, during screening, at each phase of baseline, at the postintervention assessment, and at follow-up) according to the recommendations of the Canterbury Health Laboratories (Christchurch, New Zealand). The lipid profile consisted of 5 components: total cholesterol, LDL, HDL, triglycerides, and TC/HDL ratio. Total cholesterol measures overall cholesterol level; LDL is a fat that circulates in the blood, moving cholesterol around the body where it is needed for cell repair and depositing it inside artery walls. HDL, also known as “good cholesterol,” helps remove cholesterol from the bloodstream. Triglycerides represents the main lipid component of dietary fat. The TC/HDL ratio, a sensitive predictor of heart disease [77], was also calculated.

#### Patient-Reported Outcomes

##### Daily Bowel Movement and Daily Bread Diary

Upon enrollment and throughout the study, participants were asked to complete a daily bowel movement and bread diary via an app. This app was developed by one of our research team members (Hilary Dewhurst from AgResearch, New Zealand) using a REDCap application programming interface token. The token is a unique code that is associated with a single user on a specific REDCap project, which allows the user to programmatically access data within the project. Upon installing the app by the participants, the app downloaded the diary questions from REDCap, linking the app (on that phone) to the corresponding participant’s allocated unique identifier at the point of consent to the study. All submissions were then recorded under the corresponding participant identifier. The submissions were stored on the app and submitted to REDCap with each entry. To ensure data integrity, the app “talks to” REDCap to retrieve the most recent record for the participant and increment accordingly, preventing any answers from being overwritten.

Each diary took approximately 2 minutes to complete each time. The bowel movement diary provided a comprehensive record of the bowel habits of the participants and assessed the following variables: (1) frequency of bowel motions, which included questions on spontaneity and completeness of bowel movement; (2) ease of defecation or level of straining; (3) fecal form based on the Bristol stool scale; (4) menstruation (if applicable); and (5) presence of blue dye (if applicable, see the following sections for further details).

The daily bread diary assessed compliance with the consumption of intervention bread and general bread consumption preferences. Consumption of bread slices and days consumed for each participant were determined through the following equations:

Compliance regarding bread slices consumed = ([number of bread slices consumed / number of bread slices expected to be consumed] × 100%)

Compliance regarding days consumed = ([number of days consumed / number of days expected to be consumed] × 100%)

Compliance with the intervention (bread slices and days consumed) was determined by calculating the mean percentage compliance for each bread and the proportion of participants meeting >50% compliance.

This bread diary also determined (1) whether the DRB-fortified and placebo breads were toasted; (2) minutes of toasting (if applicable); (3) additional commercial sliced bread consumed (if applicable, during the washout period); and (4) the type, brand, and amount of additional bread consumed (if applicable).

##### Web-Based Questionnaires

###### Overview

One day before each phase of baseline, postintervention, and follow-up clinic visits, participants received an individualized email with a link to 6 web-based questionnaires. These web-based questionnaires, which were emailed to participants, were similarly administered via the REDCap system. All these questionnaires took approximately 15 minutes to complete each time.

###### Gastrointestinal Symptom Rating Scale

The Gastrointestinal Symptom Rating Scale, a validated instrument that assesses gut comfort, was administered before and during the intake of intervention and placebo bread. The Gastrointestinal Symptom Rating Scale has a 1-week recall that assesses symptom severity using a 7-point Likert scale ranging from “no discomfort at all” to “very severe discomfort.” The complete instrument consists of 15 primary items clustered into 5 domains: diarrhea, constipation, reflux, abdominal pain, and indigestion [78-80].

###### Patient-Reported Outcome Measurement Information System: Anxiety and Depression

This consists of a validated system with multiple domains, where specific domains can be chosen to be integrated into diverse data collection tools. This chosen questionnaire evaluates anxiety and depression in the last 7 days in detail as well as mental symptoms rated by severity from “not at all” to “very much” and from “never” to “always” [81,82].

###### World Health Organization 5-Question Well-Being Index

This is a short self-reported rating scale of current well-being. It consists of 5 statements concerning the previous 2 weeks adjusted to 1 week (*all of the time*=5; *most of the time*=4; *more than half of the time*=3; *less than half of the time*=2; *some of the time*=1; *at no time*=0) [83].

###### Warwick-Edinburgh Mental Wellbeing Scale

A 14-item scale adjusted to 1 week was used to explore mental health and well-being over the previous 2 weeks. The Warwick-Edinburgh Mental Wellbeing Scale has 5 response categories summed to provide a single score. The items are all worded positively and cover both feeling and functioning aspects of mental well-being. This questionnaire covers key aspects of psychological functioning—optimism, autonomy, agency, curiosity, clarity of thought, and positive relationships—and positive affect (feelings)—confidence, feeling relaxed and cheerful, and having energy to spare (*none of the time*, *rarely*, *some of the time*, *often*, and *all of the time*) [84].

###### Multidimensional Fatigue Symptom Inventory–Short Form

This is a 20-item self-report instrument with a 7-point scale that indicates to what extent each statement applies to the participant, ranging from “yes, that is true” to “no, that is not true,” designed to measure fatigue. It covers the following dimensions: general fatigue, physical fatigue, mental fatigue, reduced motivation, and reduced activity [85].

###### Subjective Vitality Scale

A 6-statement instrument assesses the state of feeling alive and alert (ie, having energy available to oneself). The statements explore the current feeling on a scale of 1 to 7 (1=*not at all true*, 4=*somewhat true*, and 7=*very true*) [86].

#### Dietary Intake

Paper copies of a nonconsecutive 3-day food diary were posted to all enrolled participants to complete before each of the 5 study visits (ie, at baseline, postintervention assessment, and follow-up). All enrolled participants were provided with verbal and written instructions by the study dietitian on completing a food diary. The written information included instructions on how to describe food using household measures. The food diary included written prompts for detailed information about when and where the food was consumed and who they were with when the food was consumed; the type of food or drink; brand details; preparation or cooking methods; and quantity, as well as a section for recipe details. Participants were also asked to record any reasons why their intake may have differed from their normal eating routines. Before each subsequent appointment, participants received reminder emails to start the food diary and the same written information on completing the food diary. Participants could contact the study dietitian if they required further guidance on completing the food diary. At each clinic visit, the study dietitian reviewed each food diary to ensure that adequate detail was provided and check whether additional foods and beverages were consumed but not recorded. In total, 2 nutrition graduates trained in dietary assessment entered the food diaries into a dietary analysis software, Foodworks Online Professional (version 1.0; Xyris Software) [87]. The New Zealand Food Composition Database (FOODfiles 2018; version 1.0) was used to estimate energy and nutrient intakes. After the primary outcome was analyzed and the study was unblinded to the intervention, the macro- and micronutrients from the 2 types of study bread were manually added to the dataset based on the type of bread and actual reported bread consumption. This final dataset was then exported into SPSS (version 29; IBM Corp) and was treated as continuous data.

#### Physiome Measurements

##### Blue Food Dye

At each baseline and postintervention clinic visit, participants ingested 1 teaspoon of Royal Blue Liqua-Gel (Chefmaster) food coloring (12 drops per 1.5 grams) in water. The intake time and date were recorded. The dye was poorly absorbed or fermented, which allowed for analysis of whole-gut transit time upon passing through visual confirmation [88,89]. Visual confirmation was recorded by the participant on the daily bowel movement diary app.

##### Atmo Gas-Sensing Capsule

A subset of participants (n=15) completed an assessment of colonic gas profiling and whole-gut and regional transit time through the Atmo gas-sensing capsule. The capsule measures temperature; a range of gaseous fermentation by-products, including carbon dioxide and hydrogen; and an indication of oxygen level, capsule tumble, and antenna reflectometry [90,91]. The selected participants received a separate participant information sheet and completed a specific consent form before the transit assessment. The capsule was ingested at each phase of baseline and at the end of the postintervention clinic visits. These participants consumed a standardized cereal bar and an Atmo gas-sensing capsule (Atmo Biosciences).

The selected participants were fitted with a sling bag containing a transponder. It consists of a receiver and smartphone with a custom app that “listens” to the capsule ingested. These participants wore or kept the Atmo transponder close to them (approximately 1 m) until the exit of the capsule or for up to 5 days. They were also asked to enter additional daily bowel movement diary information into the Atmo transponder. The data were collected in real time on the smartphone app and transmitted to an Atmo cloud during the passage to allow for remote monitoring and troubleshooting. Once the participant confirmed the exit of the capsule, they returned the transponder to the research team. The diary data from the Atmo app were then synchronized to the Atmo cloud for review and analysis. Blinded and deidentified data synchronized to the Atmo cloud were assessed using specialized Atmo software by trained Atmo team members. The analysis included colonic gas profiling (hydrogen and carbon dioxide) and the determination of whole-gut and regional transit times.

##### Adverse Events

Although the study is considered low risk, all participants were provided with information about managing possible side effects. Participants were encouraged to contact their health care provider, followed by our research team, in the event of side effects. Participants were encouraged to record any symptoms and may withdraw if they experienced any side effects. Any participants experiencing harm directly due to their involvement in the study were withdrawn from the study immediately.

#### Sample Size

Sample size calculations were based on the GutFeelingKB cohort [92]. The percentage of relative abundance of a composite microbiome index (incorporating 15 operational taxonomic units as outlined in this section) was estimated to be 28.3% with an SD of 14%. The sample size needed was 60 participants in this crossover study to account for a 15% dropout rate. The crossover study had >80% power to detect a difference in the absolute increase in the relative abundance of approximately 6% as statistically significant (2-tailed α=.05). An increase of 6% compared with the baseline level of 28% equates to a relative increase of approximately 22%. The consumption of 3 (for female individuals) or 4 (for male individuals) slices of DRB-fortified bread per day for 28 days is thought to affect individual and combined groups of the microbial taxa making up the composite.

The composite microbiome index included genera from 5 phyla found in the gut microbiota of healthy human adults. This index included the *Prevotella* and *Barnesiella* genera and *Bacteroides ovatus* and *Bacteroides xylanisolvens* from the *Bacteriodetes* phylum; the *Roseburia*, *Anaerostipes*, *Blautia*, *Eubacterium*, *Ruminococcus*, *Faecalibacterium*, and *Lactobacillus* genera from the *Firmicutes* (*Bacillota*) phylum; the *Bifidobacterium* and *Eggerthella *genera from the *Actinobacteria* (*Actinomycetota*) phylum; the *Akkermansia* genera from the *Verrucomicrobiota* phylum; and the *Methanobrevibacter* genera from the *Euryarcheaota* phylum.

#### Statistical Analysis Plan

This study was conducted as a superiority trial. The guidelines of the Committee for Proprietary Medicinal Products (now termed Committee for Medicinal Products for Human Use) require intention-to-treat analysis [93]. For a crossover design, the intention-to-treat analysis means that every enrolled participant who has consumed both the intervention and placebo bread is included despite dropout or noncompliance. All data from randomized participants who received at least one intervention dose and had at least one postintervention measurement will be analyzed.

The mean differences from the baseline of most outcomes will be compared between groups and referred to as a between-group comparison. For most outcomes, within-group analysis (after the intervention compared with baseline) will also be performed. For metabolome analyses, postintervention values (not the mean differences) will be compared within and between groups.

All statistical analyses will be performed using SPSS statistics (version 29; IBM Corp) or R (version 4.3.1) by blinded researchers under the guidance of an independent biostatistician.

Baseline characteristics will be presented using descriptive statistics. The mean and SD, or median and range, will be used to describe continuous variables. Frequencies or percentages will be used to describe categorical variables. The group effects (ie, the mean change from baseline between groups) will be assessed using (parametric) 2-tailed *t* tests and (nonparametric) Mann-Whitney or Kruskal-Wallis tests for symmetrically and asymmetrically distributed data. Categorical variables will be assessed using chi-square tests (or Fisher exact tests for small samples). A 2-tailed *P*<.05 is determined as statistically significant. A value of *P*≤.10 will be considered a trend for most variables except for the microbiome data.

For the primary outcome, to determine the microbiome composite, the selected genera and species will be summed to give a composite relative abundance for each participant. Changes in the composite of the microbial taxa within and between groups will be assessed using the nonparametric Wilcoxon signed rank test. The statistical significance level is determined at *P*<.05, with a false discovery rate (FDR) of *P*<.10. A probable biological significance level (trend) is assumed at an unadjusted *P*≤.10. For other fecal microbiome measures, univariate and multivariate statistical analyses will be used to assess α- and β-diversity variations, individual relative abundance differences in taxa, and gene abundance differences between groups. Differences in α-diversity between baseline and the postintervention assessment and between groups after the intervention will be assessed using the nonparametric Wilcoxon signed rank test with continuity correction. The Benjamini-Hochberg method will be applied to control the FDR inherent in multiple-hypothesis testing for variables in the microbiome and metabolome analyses. The resulting adjusted *P* values will be computed to address the inflated type-I error rates associated with numerous comparisons. *P*<.05 and an FDR of *P*<.10 will be deemed significant, with a probable biological significant difference (trend) assumed at an unadjusted *P*≤.10.

Box plots will be created using *ggplot2* to compare groups for each Chao1 and Shannon Indexes. The permutational multivariate ANOVA and the analysis of dissimilarity functions will then be used to assess the statistical significance of microbial community composition between individuals. Analysis of dissimilarity uses permutation testing and provides statistical assessments and their significance using *F* tests based on the sequential sums of squares from the permutations to determine whether the observed differences in β-diversity are statistically meaningful. Microbial differential gene expression will be performed using quasi-likelihood *F* tests. This test provides stricter error rate control by accounting for the uncertainty in dispersion estimation. Genes with an absolute log fold change of >1.1 will be considered as differentially expressed.

As mentioned previously, the comparisons will be between each group and baseline and between groups after the intervention for plasma or fecal metabolome measures. The partial least squares projection discriminant analysis (PLS-DA) model will be performed to identify metabolites using SIMCA (version 17; Umetrics). The PLS-DA models will be subject to 100-fold permutations to evaluate performance; the models will be validated using the predictive ability of the model (Q2) and the ANOVA testing of cross-validated predictive residuals (ANOVA testing of cross-validated predictive residuals) [94]. The most discriminating features will be selected for building PLS-DA models. These models will be again subjected to prediction accuracy and overfitting assessment by the corresponding tests (ANOVA testing of cross-validated predictive residuals) and comparison of the Q2 afterward. Features will be selected based on their variable importance in the projection score. Univariate statistical analysis and heat maps will be performed using MetaboAnalyst (version 5.0) [95]. The metabolite and lipid relative intensity differences between groups will be tested through *t* tests or Wilcoxon rank sum tests. The multiple testing corrections will be controlled using FDR correction [96].

#### Data Quality and Assurance

The researchers collecting data were trained accordingly in data collection, maintaining security and health-related confidentiality.

Due to the large amount of data to be collected, there may be an inherent risk of errors during the data entry into the data bank. All paper-based data were handled by blinded study personnel, and participants were strongly encouraged to enter survey data directly into the database via email links to the surveys and via the app to minimize this risk.

The nutrition graduates who entered the raw food and beverage diary data maintained a data entry assumption spreadsheet to ensure consistency of data entry. The study dietitian will independently audit 20% of the food diaries entered into the dietary analysis program (Foodworks Online; version 1.0) by the nutrition graduates. Diaries were checked for accuracy, consistency, and completeness. Extreme values or outliers for specific nutrients were identified and clarified by reviewing the assumption spreadsheet and raw food and beverage diary data.

A formal data monitoring committee was not required as the intervention and placebo breads in this study are considered as sufficiently low risk that no harm is anticipated to occur beyond the risks of standard care and everyday life.

Data monitoring occurred through weekly meetings of the research team. The unblinded research team maintained concealment.

#### Data Collection and Storage by Researchers

Data collection for questionnaires occurred electronically via the REDCap system using the unique identifier allocated to the participants at the point of consent to the study. Participants who preferred paper data collection were accommodated. Paper-based and telephone-collected data were entered directly into electronic data collection systems (using REDCap) as soon as they were received.

Biological and physiome data were analyzed as per the aforementioned measures. The data files were stored in Microsoft Excel (Microsoft Corp) for raw data and as data analysis files (SPSS statistics version 29.0 or R statistical software version 4.3.1). These data were only identified by the unique code of each participant. Participants were identifiable to researchers only by their study code. Biological samples were stored in secure facilities with restricted access until publication of the results but not longer than 10 years, after which they will be destroyed hygienically in accordance with New Zealand Standard 4304:2002 (management of health care waste) or with the appropriate *karakia* (for Māori participants).

Raw data collected in hard copy were stored after electronic data entry as part of the case report form in a locked filing cabinet. All electronic data files generated in the study were stored on a password-secured University of Otago server or Otago OneDrive cloud storage and were accessed (and downloaded as needed) via the password-protected computers of named investigators and stored on locked premises. A master file containing participants’ personal identifying information was only accessible to the researchers involved in data collection. The blinded researchers had only access to deidentified raw data files and were responsible for the final data analysis.

## Results

Recruitment of participants began on June 1, 2022, and was completed on December 15, 2022. The study was approved by the University of Otago Human Ethics Committee (Health) on May 23, 2022. Of the 66 individuals who completed the web-based preliminary screening questionnaire and attended an on-site screening, 6% (n=4) were excluded as they did not meet the inclusion criteria. Therefore, 62 participants were randomized. During the first intervention phase, 10% (6/62) of the participants dropped out, resulting in a total of 56 participants (n=33, 59% female; n=23, 41% male; n=41, 73% New Zealand European; n=1, 2% Māori) included in the analysis.

The mean age for the entire cohort is 40.4 (SD 13.4) years, and the mean BMI is 21.9 (SD 10.3) kg/m^2^. Due to the large dataset, data analysis for all outcomes was planned to be completed by the last quarter of 2024, with full results expected to be published in peer-reviewed journals by the end of 2024.

## Discussion

### Anticipated Findings

The primary aim of this study is to determine the influence of DRB-fortified bread on the relative abundances of a composite of selected key genera and species of the gut microbiota that are relevant in DF degradation and metabolism in healthy adults with low DF intake as compared with placebo white toast bread. In addition, relative abundance or concentration of fecal or plasma biological markers of host and microbial interactions (microbial taxa, bile acids, and organic acid concentrations) alongside cardiovascular health, patient-reported outcomes, and physiome outcomes of the recruited healthy participants will be described. The findings of this study are anticipated to provide a better understanding of the habitual impact of DRB-fortified bread on the gut microbiome and all other assessed parameters in healthy individuals with low DF intake.

Preliminary analysis showed that, during the first intervention phase, 10% (6/62) of the participants dropped out, resulting in a total of 56 participants (n=41, 73% New Zealand European and n=1, 2% Māori) included in the intention-to-treat analysis. Due to the large dataset, data analysis and publication of full results are expected to be completed by the end of 2024.

This is the first human study to explore DRB-fortified bread as a dietary intervention. Therefore, it was decided to recruit a generally healthy population of male and female individuals with low DF intake before the commencement of the study. In addition, participants were given a daily dose of 3 to 4 slices of DRB-fortified or white toast bread, which could easily be added to the diet. The bread slices were provided in separate doses tailored to the nutrient requirements for female (3 slices) and male (4 slices) individuals. Consequently, these strategies to improve DF intake among low DF consumers were practical and could be implemented in real-life settings. Furthermore, participants were specifically asked about their regular bread consumption. Therefore, only individuals who consumed bread were included in the study, which may reduce the generalizability of the study results. Nonetheless, these risk management measures aimed to minimize dropout and enhance the cost-effectiveness of the study.

Fecal samples were collected to determine the gut microbiota composition, gene abundances, bile acid concentrations, organic acid concentrations, and moisture content. Immediate analysis of fresh fecal samples is often unrealistic, raising concerns about storage methods by participants, which may cause microbial DNA degradation, overgrowth, and species death in fecal samples [97]. However, there are room-temperature microbial stabilization methods with high technical reproducibility and compositional stability [98], which should be considered for future studies. From a cultural perspective, these advanced methods offer an alternative for future studies in which refrigerating fecal samples is not feasible, particularly in cases in which it may be culturally insensitive, such as for Māori and Pasifika participants. Nonetheless, all participants were given clear written instructions, ice packs, and a cooler bag for returning their samples during clinic visits, minimizing potential confounding effects.

Strengths of the Bread Related Effect on Microbial Distribution study include using shotgun metagenomics to analyze extracted fecal microbial DNA. This approach allowed for the exploration of the composition and predictive functional aspects of the gut microbiota. These analyses provided insights into the community structure and diversity as well as the identification of changes in taxa and genes associated with the study interventions [99]. In addition, this study included other multiomic approaches (metabolomics), validated subjective questionnaires, and dietary records collected before and after each intervention. Integrating these data will allow for a better understanding of the complex interactions among diet, gut microbiota, and other health outcomes in healthy adults with low DF intake. Furthermore, this study is a blinded crossover study, allowing participants to be their own controls [47]. A crossover design was chosen due to the recognized inter- and intraindividual variabilities among populations, allowing for a reduced sample size while achieving the required statistical power. In addition, sequence effects were accounted for in the analysis [100].

Future studies may consider recruiting individuals with gut abnormalities, such as individuals with constipation, or conduct further subgroup analysis on the same dataset to separate individuals with different BMI statuses and gut microbiota compositions and of different ethnicities at baseline. These considerations may provide a better understanding of the effects of DRB-fortified bread on all the assessed study outcomes.

In addition, there is also a possibility that participants felt obligated to consume the interventions provided considering the perceived notion of being part of research. Future studies should consider supplementing the study with qualitative interviews or surveys. Including consumer insights as part of the study would allow for a better understanding of participants’ perspectives and experiences. Subsequently, this may allow for further generalization to the general population should DRB-fortified bread be introduced to the market.

### Conclusions

In this double-blinded, crossover randomized controlled trial, the collection of diet, clinical, biological (microbiota, metabolites, and lipids), and physiome (transit time and gas profile) data will provide crucial information on the benefits of fortifying DRB in commonly consumed foods. This knowledge may help support the public health system and food industry in developing targeted interventions aimed at modulating health-promoting gut microbes, general and mental well-being, DF intake, whole-gut and regional transit time, and fermentation gas profiles in healthy adults with low DF intake.

## Appendix

Multimedia Appendix 1 SPIRIT Outcomes 2022 Checklist.

## Abbreviations

bp: base pairs
CONSORT: Consolidated Standards of Reporting Trials
DF: dietary fiber
DRB: defatted rice bran
eCRF: electronic case report form
FDR: false discovery rate
HDL: high-density lipoprotein
HPLC: high-pressure liquid chromatography
LCMS: liquid chromatography mass spectrometry
LDL: low-density lipoprotein
MS: mass spectrometry
PLS-DA: partial least squares projection discriminant analysis
REDCap: Research Electronic Data Capture
SF-12v2: 12-item Short-Form Health Survey version 2
SPIRIT: Standard Protocol Items: Recommendations for Interventional Trials
